# Supplementary figures and images for: Structural insights and characterization of human Npas4 protein
Source: PeerJ. 2018 Jun 14;6:e4978. doi: 10.7717/peerj.4978 (PMC6004298; doi:10.7717/peerj.4978)

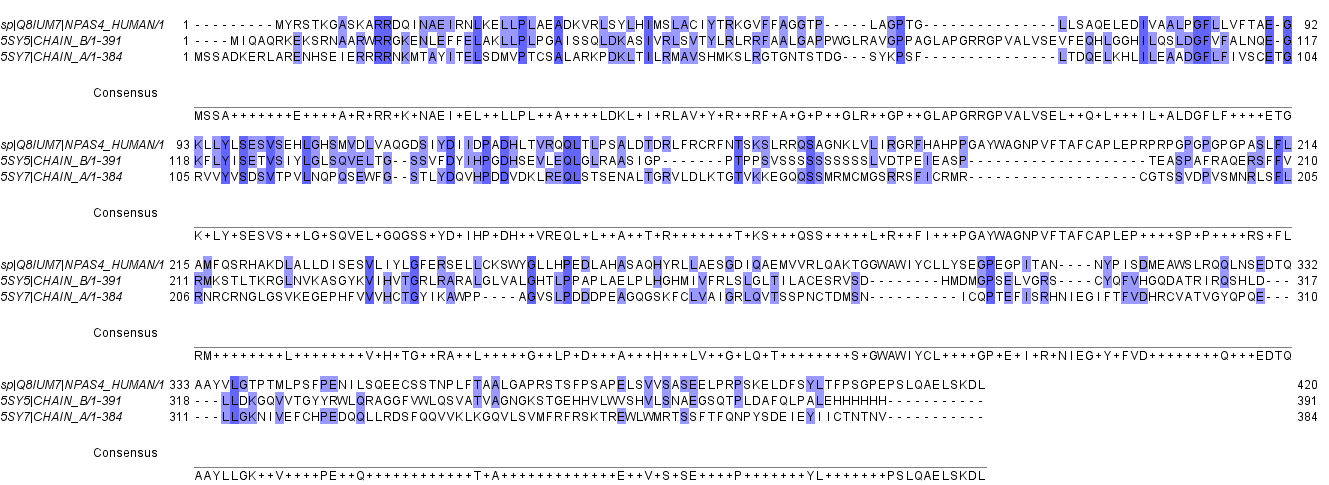

Supplement: Figure S1 — The homology is shown in blue color. [file peerj-06-4978-s001.png]

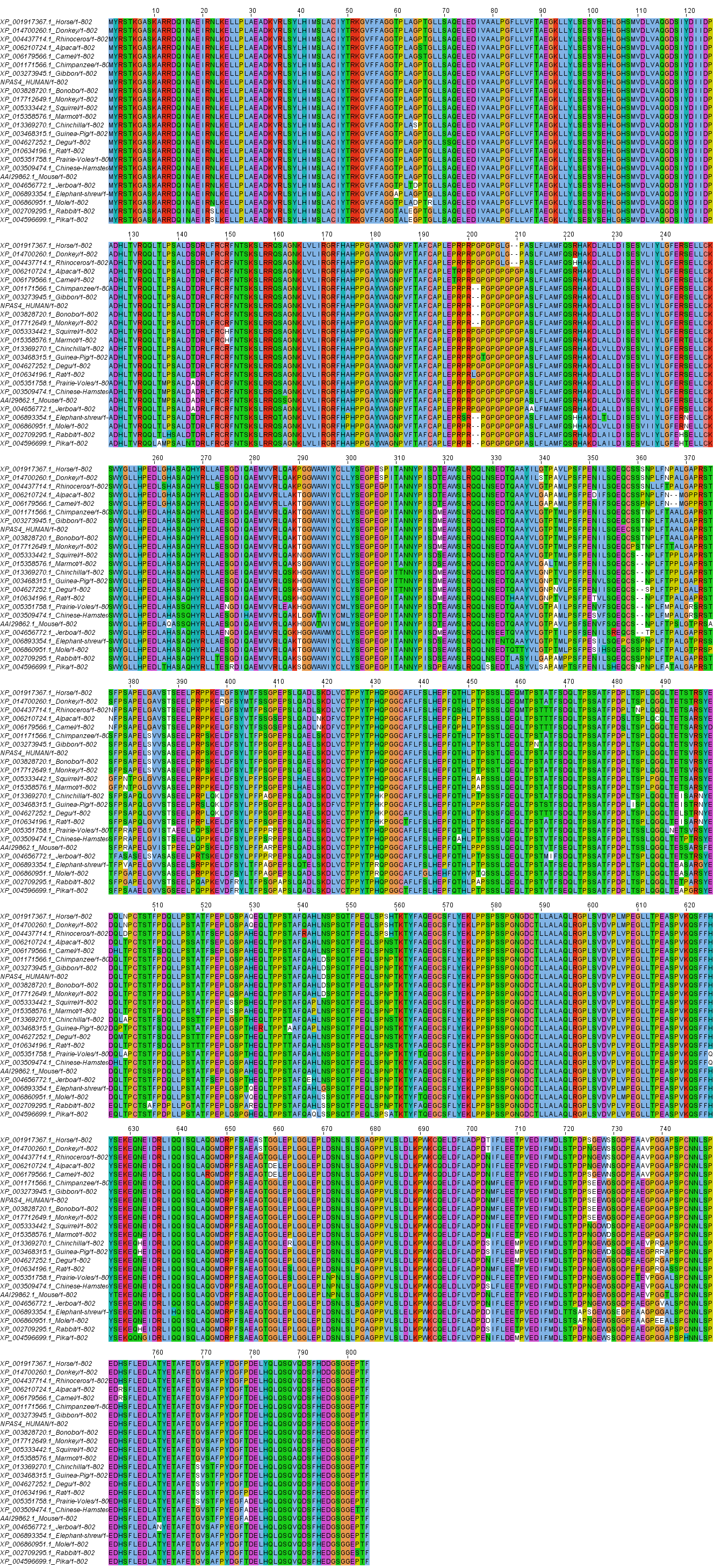

Supplement: Figure S2 [file peerj-06-4978-s002.png]

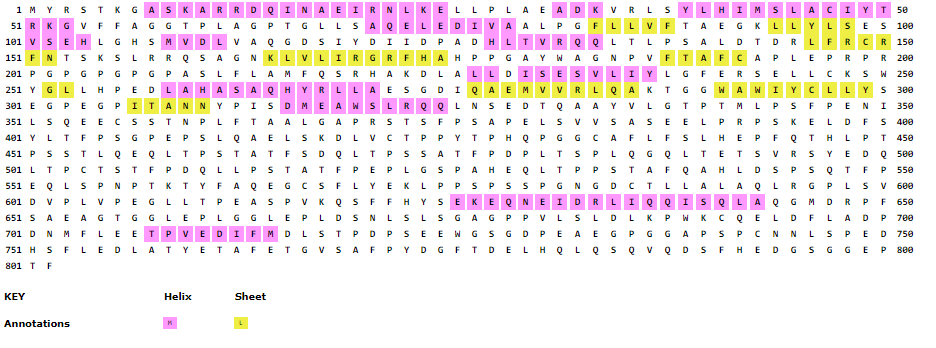

Supplement: Figure S3 [file peerj-06-4978-s003.png]
