# Supplementary material for: Structural insights and characterization of human Npas4 protein
Source: PeerJ. 2018 Jun 14;6:e4978. doi: 10.7717/peerj.4978 (PMC6004298; doi:10.7717/peerj.4978)
Supplement: Table S1 [file peerj-06-4978-s004.docx]

**Table S1:** The names, Accession No and taxonomic classification of 24 different organisms whose protein sequences are used for multiple sequence alignment and phylogenetic analysis.

| **No.** | **NCBI Accession No.** | **Organism** | **Taxonomic Classification** |
| --- | --- | --- | --- |
|  | Q8IUM7 | Homo Sapiens (Human) | \| Kingdom: \| Animalia \| \| --- \| --- \| \| Phylum: \| Chordata \| \| Class: \| Mammalia \| \| Order: \| Primates \| |
|  | XP_001171566 | Pan troglodytes (Chimpanzee) | \| Kingdom: \| Animalia \| \| --- \| --- \| \| Phylum: \| Chordata \| \| Class: \| Mammalia \| \| Order: \| Primates \| |
|  | XP_003828720 | [Pan paniscus](https://www.ncbi.nlm.nih.gov/Taxonomy/Browser/wwwtax.cgi?id=9597)  (Bonobo) | \| Kingdom: \| Animalia \| \| --- \| --- \| \| Phylum: \| Chordata \| \| Class: \| Mammalia \| \| Order: \| Primates \| |
|  | XP_003273945 | Nomascus leucogenys  (northern white-cheeked gibbon) | \| Kingdom: \| Animalia \| \| --- \| --- \| \| Phylum: \| Chordata \| \| Class: \| Mammalia \| \| Order: \| Primates \| |
|  | XP_017712649 | Rhinopithecus bieti (black snub-nosed monkey) | \| Kingdom: \| Animalia \| \| --- \| --- \| \| Phylum: \| Chordata \| \| Class: \| Mammalia \| \| Order: \| Primates \| |
|  | XP_004437714 | Ceratotherium simum simum  (southern white rhinoceros) | \| Kingdom: \| Animalia \| \| --- \| --- \| \| Phylum: \| Chordata \| \| Class: \| Mammalia \| \| Order: \| Perissodactyla \| |
|  | XP_001917367 | Equus caballus  (horse) | \| Kingdom: \| Animalia \| \| --- \| --- \| \| Phylum: \| Chordata \| \| Class: \| Mammalia \| \| Order: \| Perissodactyla \| |
|  | XP_014700260 | Equus asinus  (Donkey) | \| Kingdom: \| Animalia \| \| --- \| --- \| \| Phylum: \| Chordata \| \| Class: \| Mammalia \| \| Order: \| Perissodactyla \| |
|  | XP_015358576 | Marmota marmota marmota  (Alpine marmot) | \| Kingdom: \| Animalia \| \| --- \| --- \| \| Phylum: \| Chordata \| \| Class: \| Mammalia \| \| Order: \| Rodentia \| |
|  | XP_006893354 | Elephantulus edwardii (Cape elephant shrew) | \| Kingdom: \| Animalia \| \| --- \| --- \| \| Phylum: \| Chordata \| \| Class: \| Mammalia \| \| Order: \| Macroscelidea \| |
|  | XP_004627252 | Octodon degus  (degu) | \| Kingdom: \| Animalia \| \| --- \| --- \| \| Phylum: \| Chordata \| \| Class: \| Mammalia \| \| Order: \| Rodentia \| |
|  | XP_010634196 | Fukomys damarensis (Damara mole-rat) | \| Kingdom: \| Animalia \| \| --- \| --- \| \| Phylum: \| Chordata \| \| Class: \| Mammalia \| \| Order: \| Rodentia \| |
|  | XP_004656772 | Jaculus jaculus  (lesser Egyptian jerboa) | \| Kingdom: \| Animalia \| \| --- \| --- \| \| Phylum: \| Chordata \| \| Class: \| Mammalia \| \| Order: \| Rodentia \| |
|  | XP_002709295 | Oryctolagus cuniculus (rabbit) | \| Kingdom: \| Animalia \| \| --- \| --- \| \| Phylum: \| Chordata \| \| Class: \| Mammalia \| \| Order: \| Lagomorpha \| |
|  | XP_013369270 | Chinchilla lanigera  (long-tailed chinchilla) | \| Kingdom: \| Animalia \| \| --- \| --- \| \| Phylum: \| Chordata \| \| Class: \| Mammalia \| \| Order: \| Rodentia \| |
|  | XP_006860951 | Chrysochloris asiatica  (Cape golden mole) | \| Kingdom: \| Animalia \| \| --- \| --- \| \| Phylum: \| Chordata \| \| Class: \| Mammalia \| \| Order: \| Afrosoricida \| |
|  | XP_005351758 | Microtus ochrogaster (prairie vole) | \| Kingdom: \| Animalia \| \| --- \| --- \| \| Phylum: \| Chordata \| \| Class: \| Mammalia \| \| Order: \| Rodentia \| |
|  | XP_006210724 | Vicugna pacos  (alpaca) | \| Kingdom: \| Animalia \| \| --- \| --- \| \| Phylum: \| Chordata \| \| Class: \| Mammalia \| \| Order: \| Artiodactyla \| |
|  | XP_006179566 | Camelus ferus  (Wild Bactrian camel) | \| Kingdom: \| Animalia \| \| --- \| --- \| \| Phylum: \| Chordata \| \| Class: \| Mammalia \| \| Order: \| Artiodactyla \| |
|  | AAI29862 | Mus musculus  (house mouse) | \| Kingdom: \| Animalia \| \| --- \| --- \| \| Phylum: \| Chordata \| \| Class: \| Mammalia \| \| Order: \| Rodentia \| |
|  | XP_003468315 | Cavia porcellus  (domestic guinea pig) | \| Kingdom: \| Animalia \| \| --- \| --- \| \| Phylum: \| Chordata \| \| Class: \| Mammalia \| \| Order: \| Rodentia \| |
|  | XP_003509474 | Cricetulus griseus  (Chinese hamster) | \| Kingdom: \| Animalia \| \| --- \| --- \| \| Phylum: \| Chordata \| \| Class: \| Mammalia \| \| Order: \| Rodentia \| |
|  | XP_004596699 | Ochotona princeps (American pika) | \| Kingdom: \| Animalia \| \| --- \| --- \| \| Phylum: \| Chordata \| \| Class: \| Mammalia \| \| Order: \| Ochotonidae \| |
|  | XP_005333442 | Ictidomys tridecemlineatus (thirteen-lined ground squirrel) | \| Kingdom: \| Animalia \| \| --- \| --- \| \| Phylum: \| Chordata \| \| Class: \| Mammalia \| \| Order: \| Rodentia \| |
