# Supplementary material for: Structural insights and characterization of human Npas4 protein
Source: PeerJ. 2018 Jun 14;6:e4978. doi: 10.7717/peerj.4978 (PMC6004298; doi:10.7717/peerj.4978)
Supplement: Table S2 [file peerj-06-4978-s005.docx]

| **Name of residue** | **Position** | **Kinase** |
| --- | --- | --- |
| Serine | 4 | PKC |
|  | 9 | PKC |
|  | 38 | PKC,PKA |
|  | 44 | DNAPK, PKA |
|  | 70 | CKII |
|  | 139 | CDC2, PKG |
|  | 154 | CDC2 |
|  | 156 | PKC,CDC2 |
|  | 161 | PKA, PKG, RSK |
|  | 273 | CKII |
|  | 315 | CKII |
|  | 321 | PKC |
|  | 328 | CKII, CDC2 |
|  | 345 | PKG |
|  | 352 | ATM, DNAPK, CKII |
|  | 374 | RSK, PKC |
|  | 382 | CDC2 |
|  | 385 | CKII |
|  | 394 | PKA |
|  | 496 | CKI |
|  | 546 | DNAPK, CDC2 |
|  | 577 | CDK5, P38MAPK |
|  | 673 | CKI |
|  | 785 | PKC, ATM |
| Threonine | 5 | PKC |
|  | 60 | P38MAPK |
|  | 130 | PKC |
|  | 136 | PKC |
|  | 143 | PKC |
|  | 153 | PKB, PKG |
|  | 331 | DNAPK |
|  | 339 | P38MAPK |
|  | 359 | CDC2 |
|  | 403 | PKC, CKI |
|  | 427 | CDK5, P38MAPK |
|  | 611 | P38MAPK, CDK5 |
|  | 728 | CKII |
| Tyrosine | 401 | INSR |
